# Predictors of the timing of initiation of antenatal care in an ethnically diverse urban cohort in the UK

**DOI:** 10.1186/1471-2393-13-103

**Published:** 2013-05-03

**Authors:** Jenny A Cresswell, Ge Yu, Bethan Hatherall, Joanne Morris, Farah Jamal, Angela Harden, Adrian Renton

**Affiliations:** 1 London School of Hygiene and Tropical Medicine, London, United Kingdom; 2 University of Leeds, Leeds, United Kingdom; 3 Institute for Health and Human Development (IHHD), University of East London, London, United Kingdom; 4 Barts Health NHS Trust, London, United Kingdom

## Abstract

**Background:**

In the UK, women are recommended to engage with maternity services and establish a plan of care prior to the 12th completed week of pregnancy. The aim of this study was to identify predictors for late initiation of antenatal care within an ethnically diverse cohort in East London.

**Methods:**

Cross-sectional analysis of routinely collected electronic patient record data from Newham University Hospital NHS Trust (NUHT). All women who attended their antenatal booking appointment within NUHT between 1st January 2008 and 24th January 2011 were included in this study. The main outcome measure was *late antenatal booking*, defined as attendance at the antenatal booking appointment after 12 weeks (+6 days) gestation. Data were analysed using multivariable logistic regression with robust standard errors.

**Results:**

Late initiation of antenatal care was independently associated with non-British (White) ethnicity, inability to speak English, and non-UK maternal birthplace in the multivariable model. However, among those women who both spoke English and were born in the UK, the only ethnic group at increased risk of late booking were women who identified as African/Caribbean (aOR: 1.40: 95% CI: 1.11, 1.76) relative to British (White). Other predictors identified include maternal age younger than 20 years (aOR: 1.32; 95% CI: 1.13-1.54), high parity (aOR: 2.09; 95% CI: 1.77-2.46) and living in temporary accommodation (aOR: 1.71; 95% CI: 1.35-2.16).

**Conclusions:**

Socio-cultural factors in addition to poor English ability or assimilation may play an important role in determining early initiation of antenatal care. Future research should focus on effective interventions to encourage and enable these minority groups to engage with the maternity services.

## Background

In the UK, women are recommended to engage with maternity services and establish a plan of care prior to the 12th completed week of pregnancy [1]. Although there has been little rigorous evaluation of optimal timing of the first antenatal appointment [2], delayed initiation of antenatal care limits the time available for women to consider and make informed decisions regarding their care. Late initiation of antenatal care may preclude the offer of an early ultrasound scan for accurate gestational age assessment. Nutritional supplements, such as folic acid, need to be taken very early in pregnancy in order to be effective; and women should be given advice regarding appropriate diet and exercise, the consequences of smoking and alcohol consumption during pregnancy as early as possible [3].

The antenatal “booking appointment” should take place at 8–12 weeks gestation and may take place in either a hospital or in the community. The booking appointment is the time at which a detailed history is taken, usually by a midwife, and represents the first opportunity to check a woman’s ‘risk status’; women should be screened for risk factors for gestational diabetes and pre-eclampsia at the booking appointment so that an appropriate monitoring and care plan can be implemented in good time where these are identified [3].

A key justification for the prompt referral of women to maternity services is to provide the opportunity to make an informed choice concerning all available antenatal screening options. Screening for infectious diseases, including HIV, syphilis and hepatitis B, should take place as early in pregnancy as possible [4], in order to allow appropriate interventions to be offered. The optimum time for screening for haemoglobin disorders (sickle cell disease and thalassaemia) is prior to 10 weeks gestation [5]. Screening for Trisomy 21 (Down’s syndrome) should take place between 10–20 weeks gestation, ideally prior to 13 weeks +6 days gestation [5]. It is of particular concern if a woman books later than 20 weeks gestation, as she may also miss the window for the detailed fetal anomaly ultrasound scan.

Late initiation of antenatal care has been found to be associated with non-White ethnicity in several previous studies conducted in Western European settings [6-9]. A cross-sectional survey of 200 hospitals across England, administered via a postal questionnaire, found that women who identified themselves as Black or Asian were less likely than women who identified themselves as White to attend their antenatal booking appointment before 12 weeks gestation in a univariate analysis, and that being born outside the UK was strongly associated with late booking in both the univariate and multivariable models [7]. However, non-White ethnicities had a poorer response rate than women who identified themselves as White (41% versus 63%) potentially biasing the results; and there were very small numbers in several of the ethnic subgroups, limiting the statistical power of the study.

The latest Confidential Enquiries into Maternal Deaths in the UK identified pregnant migrants who may not be familiar with British language or culture as potentially vulnerable and that extra efforts should be made to book these women into maternity care with adequate translation and support [10]. Knowledge of any ethnic variations in the initiation of antenatal care is important to allow the development of targeted and appropriate interventions to encourage early contact with the maternity services.

We conducted a mixed methods study [11], set in an ethnically diverse cohort in Newham, East London, which aimed to explore the barriers to, and facilitators of, early and consistent access to antenatal care among women, through: 1) analysis of routinely collected data on antenatal care services; 2) seeking the experiences and perspectives of women and health professionals; 3) and reviewing the international research literature. Newham is one of the most ethnically diverse boroughs in the UK with 61% of the population belonging to ethnic minority groups. Newham was ranked to be the seventh most deprived local authority area in England on the 2007 Index of Multiple Deprivation [12]. The borough has the highest fertility rate in England with more than 5000 live births per year, and also has one of the highest infant mortality rates in the country (6.9 per 1000 live births compared to 5.1 per 1000 live births nationally) [12]. There is one hospital within Newham University Hospital NHS Trust (NUHT) with one maternity service. Antenatal care is delivered both in hospital and in general practices by community midwives, although the initial booking appointment usually takes place centrally in the hospital. In NUHT, the majority of women are referred to their booking appointment following a brief initial consultation with their general practitioner (GP); however women can also contact the maternity services directly and complete a self-referral form. Antenatal care is midwife-led, with referral to higher-level specialists as necessary.

This paper reports on the quantitative component of the wider study, the objective of which is to identify predictors of late antenatal booking. We had a particular interest in maternal ethnicity as a predictor of late antenatal booking; in this paper we investigate the associations between ethnicity and timing of access to antenatal care in greater detail than has been conducted previously.

## Methods

This study used routinely collected electronic patient record data from women who attended their antenatal booking appointment between 1st January 2008 and 24th January 2011 within NUHT, thus representing all women who sought care maternity care in the public sector during this period. In total 20,177 women attended their booking appointment during the study period. Forty-two (0.2%) women were excluded because they did not have a valid gestational age at booking recorded, leaving a sample of 20,135 for analysis.

Gestational age at booking was calculated according to the woman’s reported last menstrual period. Where women either did not know, or were uncertain of the date of their last menstrual period, then gestational age at booking was estimated from an ultrasound scan. The outcome variable was late antenatal booking, defined as where the booking appointment was attended later than 12 weeks (+6 days) gestation.

Our primary exposure was maternal ethnicity, as self-defined by the woman. Other predictors investigated were maternal age, parity, maternal occupation, English language ability, maternal birthplace (UK versus non-UK) and housing status. All variables were self-reported. Some information, such as maternal birthplace and occupation, were originally recorded by the midwife as a free text field; this was subsequently coded into categorical variables. Maternal occupation was coded using an adapted version of the National Statistics Socio-Economic Classification system. Other variables were categorised as appear in Table 1. The multivariable models were also adjusted for year of the booking appointment.

**Table 1:** Description of the sample; unadjusted and adjusted odds ratios for the effect of each risk factor on late antenatal booking

**Risk factor**		**n**	**%**	**Unadjusted analysis**		Adjusted analysis *	
				**n = 20,135**		**n = 19,316**	
				**OR**	**[95% CI]**	**OR**	**[95% CI]**
Self-Defined Ethnicity	British (White)	3,875	19.25	1.00		1.00	
	African (except Somali)	2,715	13.48	2.03	[1.83, 2.25]	1.56	[1.38, 1.77]
	Somali	480	2.38	3.11	[2.56, 3.77]	1.58	[1.28, 1.96]
	Caribbean	591	2.94	1.44	[1.20, 1.73]	1.33	[1.09, 1.61]
	Eastern European	2,531	12.57	1.90	[1.71, 2.11]	1.49	[1.32, 1.69]
	Other White	395	1.96	1.34	[1.08, 1.67]	1.04	[0.82, 1.32]
	Bangladeshi (inc British)	2,867	14.24	1.55	[1.40, 1.72]	1.20	[1.07, 1.34]
	Indian (inc British)	1,842	9.15	1.34	[1.19, 1.51]	1.11	[0.97, 1.26]
	Pakistani (inc. British)	3,109	15.44	1.35	[1.22, 1.50]	1.16	[1.04, 1.29]
	Other Asian	957	4.75	1.58	[1.37, 1.83]	1.16	[0.97, 1.40]
	Other	685	3.4	1.64	[1.38, 1.94]	1.16	[0.98, 1.36]
	Missing values	88	0.44				
Age at booking (years)	<20	980	4.87	1.37	[1.20, 1.58]	1.32	[1.13, 1.54]
	20-24	4,578	22.74	1.00		1.00	
	25-29	7,137	35.45	0.73	[0.67, 0.79]	0.75	[0.69, 0.82]
	30-34	4,811	23.89	0.71	[0.65, 0.77]	0.72	[0.65, 0.79]
	35-39	2,070	10.28	0.75	[0.68, 0.84]	0.68	[0.60, 0.78]
	≥40	551	2.74	0.83	[0.69, 0.99]	0.71	[0.58, 0.88]
	Missing values	8	0.04				
Parity	No previous live births	9,815	48.75	1.10	[1.02, 1.18]	0.99	[0.92, 1.07]
	1 previous live birth	5,517	27.4	1.00		1.00	
	2 previous live births	2,763	13.72	1.14	[1.04, 1.25]	1.17	[1.06, 1.30]
	3 previous live births	1,167	5.8	1.34	[1.18, 1.52]	1.43	[1.24, 1.64]
	4+ previous live births	854	4.24	2.06	[1.78, 2.39]	2.09	[1.77, 2.46]
	Missing values	19	0.09				
Woman’s occupation	Managerial/professional	2,444	12.14	1.00		1.00	
	Lower supervisory/technical	2,492	12	1.12	[0.99, 1.27]	1.01	[0.88, 1.15]
	Semi-routine & routine	3,319	16.48	1.59	[1.42, 1.79]	1.15	[1.02, 1.31]
	Self-employed	177	0.88	2.22	[1.63, 3.02]	1.52	[1.09, 2.11]
	Housewife	6,968	34.61	1.85	[1.67, 2.05]	1.25	[1.11, 1.41]
	Unclassifiable	4,735	23.52	2.34	[2.10, 2.60]	1.59	[1.42, 1.80]
	Missing values	0	0				
Language ability	English speaker	13,466	66.88	1.00		1.00	
	Non-English speaker	6,539	32.48	1.51	[1.42, 1.60]	1.39	[1.29, 1.50]
	Missing values	130	0.65				
Birthplace	Born in the UK	4,464	22.17	1.00		1.00	
	Born outside the UK	15,666	77.8	1.52	[1.41, 1.63]	1.33	[1.21, 1.46]
	Missing values	5	0.02				
Housing status	Owner-occupier	3,752	18.63	1.00		1.00	
	Rented	14,060	69.83	1.65	[1.52, 1.78]	1.36	[1.25, 1.48]
	Temporary accommodation	344	1.71	2.54	[2.03, 3.18]	1.71	[1.35, 2.16]
	Other	1,281	6.36	2.51	[2.20, 2.86]	1.94	[1.68, 2.22]
	Missing values	698	3.47				
Year of Booking	2008	6,476	32.16	1.00		1.00	
	2009	6,450	32.03	0.60	[0.56, 0.65]	0.62	[0.57, 0.66]
	2010	6,775	33.65	0.45	[0.42, 0.49]	0.48	[0.44, 0.52]
	2011	434	2.16	0.50	[0.41, 0.62]	0.53	[0.44, 0.66]
	Missing values	0	0				

*OR*, Odds ratio; *CI*, Confidence interval.

* Each variable adjusted for all other variables in the model.

Data were cleaned and checked for consistency and potential errors. Initial tabulations and bivariate analyses were conducted for each variable to explore associations in the data. Subsequently multivariable logistic regression was used to build the final model. We used the likelihood ratio test to assess evidence of interaction between maternal ethnicity, place of birth (UK/non-UK) and ability to speak English. Stratum-specific odds ratios were subsequently calculated. Since some women (16%) had more than one pregnancy during the study period, robust standard errors were used to allow for the correlation between multiple pregnancies to the same woman.

All analysis was conducted using Stata 11.2.

## Results

**Figure 1:**
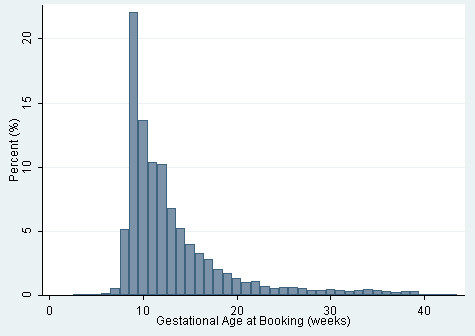
Histogram of gestational age at booking (in weeks).

Of the 20,135 women included in the analysis, 12,538 (62.5%) booked prior to the 12 weeks (+ 6 days) target, 5,089 (25.4%) women booked between 13 weeks and 19 weeks (+6 days), and 2,420 (12.1%) women booked later than 20 weeks gestation. The distribution of gestational age at booking is displayed in Figure 1.

Socio-demographic descriptive statistics for the sample are presented in Table 1. In the unadjusted analysis, all ethnic groups were significantly more likely to have their booking appointment late compared to women who identified as British-White. The timing of women’s booking appointment stratified by maternal ethnicity is presented in Table 2. Women who identified as Somali ethnicity had the highest proportion of late bookers with less than half (44.6%) booking within the 12 weeks (+6 days) target and 20.2% booking later than 20 weeks gestation. Women who identified as non-Somali African or Eastern European also tended to be late initiators of antenatal care.

**Table 2:** Gestational age at booking stratified by maternal ethnicity

**Self-defined ethnicity**	**Gestational age at booking (weeks)**				**Total**
	**Median**	**Up to 12**	**13 to 19**	**20 or more**	
	**(LQ, UQ)**	**(+6 days)**	**(+6 days)**		
British (White)	10	2,768	735	372	3,875
	(9, 13)	71.4%	19.0%	9.6%	
African	12	1,499	802	414	2,715
(except Somali)	(10, 16)	55.2%	29.5%	15.2%	
Somali	13	214	169	97	480
	(10, 18)	44.6%	35.2%	20.2%	
Caribbean	11	375	157	59	591
	(9, 14)	63.5%	26.6%	10.0%	
Eastern European	12	1,438	699	394	2,531
	(9, 16)	56.8%	27.6%	15.6%	
Other White	11	257	94	44	395
	(9, 14)	65.1%	23.8%	11.1%	
Bangladeshi	11	1,769	794	304	2,867
(incl. British)	(9, 15)	61.7%	27.7%	10.6%	
Indian	11	1,200	460	182	1,842
(incl. British)	(9, 14)	65.1%	25.0%	9.9%	
Pakistani	11	2,018	743	348	3,109
(incl. British)	(9, 14)	64.9%	23.9%	11.2%	
Other Asian	12	586	251	120	957
	(10, 15)	61.2%	26.2%	12.5%	
Other	12	414	185	86	685
	(10, 15)	60.4%	27.0%	12.6%	
**Overall**	**11**	**12,538**	**5,089**	**2,420**	**20,047**
	**(9,15)**	**62.5%**	**25.4%**	**12.1%**	

*LQ*, Lower (25^th^) quartile; *UQ*, upper (75^th^) quartile.

The results of the multivariable model are presented in Table 1. Maternal ethnicity, the ability to speak English and the mother’s place of birth were independent predictors for late initiation of antenatal care. The magnitude of effect is greatest among women who identified as Somali (aOR: 1.58; 95% CI: 1.28-1.96) or non-Somali African (aOR: 1.56; 95% CI: 1.38-1.77), compared to the White-British baseline.

The association between maternal age and initiation of antenatal care was similar in the unadjusted and adjusted analyses, with older women less likely to book late for antenatal care compared to a baseline of women aged 20–24 years. Nulliparous women were no more likely to book late than women with one previous live birth, after adjustment for age and other socio-demographic characteristics (aOR: 0.99; 95% CI: 0.92-1.07). Women with four or more previous births had over twice the odds of having their antenatal booking appointments late, compared to women with one previous birth (aOR: 2.09; 95% CI: 1.77-2.46); this was one of the strongest predictors for late antenatal booking in the multivariable model. Women who were self-employed, employed in semi-routine or routine occupations or housewives were more likely to book late, although the magnitude of the effect was attenuated in the multivariable model. Living in temporary accommodation was a strong risk factor for late antenatal booking (aOR: 1.71; 95% CI: 1.35-2.16).

There was evidence of interaction between maternal ethnicity, place of birth (UK/non-UK) and English language ability (p = 0.013) so stratum-specific odds ratios were calculated (Table 3). Among women who both spoke English and were born in the UK, the only ethnic group at increased risk of late booking were women who identified as African/Caribbean (aOR: 1.40: 95% CI: 1.11, 1.76) relative to British (White).

**Table 3:** **Effect of ethnicity on late antenatal booking, stratified by English language ability and UK/non-UK birth place**^**#**^

**Self- defined ethnicity**	**English language ability & Place of birth**	**n**	OR *	**[95% CI]**
British (White)	Speak English & born in the UK	1,662	1.00	
	Speak English & not born in the UK	919	1.02	[0.84, 1.24]
	Do not speak English & not born in the UK	1,060	1.47	[1.23, 1.76]
Eastern European or Other White	Speak English & born in the UK	532	0.88	[0.69, 1.11]
	Speak English & not born in the UK	1,199	1.42	[1.21, 1.68]
	Do not speak English & not born in the UK	1,108	3.10	[2.63, 3.66]
African or Caribbean	Speak English & born in the UK	420	1.40	[1.11, 1.76]
	Speak English & not born in the UK	2,526	1.85	[1.62, 2.12]
	Do not speak English & not born in the UK	687	2.20	[1.82, 2.66]
South Asian (Bangladeshi, Indian or Pakistani)	Speak English & born in the UK	1,196	1.14	[0.96, 1.35]
	Speak English & not born in the UK	3,549	1.25	[1.10, 1.43]
	Do not speak English & not born in the UK	2,729	2.20	[1.82, 2.66]
Other Asian	Speak English & born in the UK	48	0.94	[0.48, 1.83]
	Speak English & not born in the UK	480	1.70	[1.37, 2.11]
	Do not speak English & not born in the UK	399	1.47	[1.16, 1.85]
Other	Speak English & born in the UK	263	1.13	[0.85, 1.50]
	Speak English & not born in the UK	224	1.47	[1.09, 1.97]
	Do not speak English & not born in the UK	167	1.95	[1.41, 2.71]

*OR*, Odds ratio; *CI*, Confidence interval.

* Adjusted for maternal age at booking, parity, maternal occupation, housing status & year of booking.

^# ^Stratum-specific odds ratios were not calculated for women who did not speak English and were born in the UK, as there were only 183 women total in this sub-group.

## Discussion

Overall the proportion of women having their antenatal booking appointments late (after 12 weeks +6 days gestation) was 37.5%; with 12.1% of the total booking later than 20 weeks gestation. This is slightly higher than the NHS national average in England of 32.5% of women booking later than 12 completed weeks of gestation in the 2010–11 financial year [13]. In certain ethnic groups the proportion of women having their antenatal booking appointments late was much higher, particularly among women who identified as Somali (55.4%), non-Somali African (44.7%) or Eastern European (43.2%).

In accordance with previous studies [6-8], our analysis showed that timing of the antenatal booking appointment varied according to maternal ethnicity; however we found that the effect was modified by English language ability and place of birth. This suggests that a combination of language barriers and unfamiliarity with the maternity services, and cultural factors may play important contributing roles in women’s timing of access to antenatal care. This was explored in a concurrent qualitative study to understand the barriers to access [11]; for example, Somali and Bengali women were concerned that antenatal care would be over-medicalised and thus interfere with what was perceived to be a natural process, whilst Eastern European women felt that the process was not medicalised enough. Such findings are likely to have important consequences for the design of culturally-sensitive, patient-centred and appropriate interventions targeting these groups.

One of the key reasons for prompt antenatal booking is to allow for an informed choice regarding available antenatal screening options to be made. Infants born to African and Caribbean women, identified as groups at risk of late booking in our study, are at particular risk of haemoglobin disorders, such as sickle cell anaemia [14]. African and South Asian women are known to be at increased risk of developing gestational diabetes [15]. In areas with relatively high HIV prevalence, such as Newham [16], early access to antenatal care is important in order to offer women timely interventions to reduce the risk of mother to child transmission.

Other significant predictors for late antenatal booking were young maternal age, and indicators of potential social vulnerability such as living in temporary accommodation. Having four or more previous births was the strongest risk factor for late initiation of antenatal care (aOR: 2.09; 95% CI: 1.77-2.46); this may be because women with more experience of pregnancy are more confident and may not feel the need to initiate antenatal care so early in pregnancy, especially when they have other priorities such as childcare to consider.

Maternal ethnicity was based upon self-definition in our study. It therefore primarily represents a social and cultural construct, rather than having a biological or geographic basis. Self-reported ethnicity is known to be a poor proxy for genetic make-up [17]. It is, for example, notable that a number of women who identified themselves as British (White) yet were not able to speak English nor were born in the UK; these women may have gained citizenship through marriage or via parental descent. However, the aim of this study was to look at predictors of health-seeking behaviour which are likely to be primarily determined by socio-cultural background and life experiences, relevant to self-defined ethnicity.

Our study has a number of limitations. Data were based on self-report; there is a risk of reporting bias among women who did not speak English if adequate translation services were not available. Furthermore, there was a suggestion from the qualitative study [11] that women who identified as Somali may purposively report their last menstrual period as later that it truly was, in attempt to avoid induction of pregnancy. We did not find any statistically significant evidence of women who identified as Somali being more likely to have their reported last menstrual period being corrected after ultrasound (data not shown); however, if this did occur then it would mean that the true difference in late booking was under-estimated in our study.

As this study used routinely collected data, we were restricted in our analyses to available variables. We did not have information available on some potentially important risk factors; for example maternal education is known to be an important predictor of utilisation of health care. Some women may have initiated aspects of maternity care, such as smoking or alcohol cessation, prior to the booking appointment, particularly if the pregnancy was planned. Parts of the booking form completed by the midwife, such as maternal place of birth and occupation, were stored in a free-text format. This has implications for data validity as the information was not always recorded with sufficient clarity and detail; for example, over 20% of women had an “unclassifiable” occupation. Furthermore, recoding the free-text fields into a usable format was resource-intensive process.

Missing data in this study were relatively low (just 3.6% of the sample had a missing value on one or more covariate). However, it should be noted that the most recent Confidential Enquiries report into maternal deaths in the UK (CMACE) observed that many of the women who died during the 2006–2008 period had insufficient information recorded during the booking appointment to define their ‘risk status’ [10] and it is, therefore, important to be aware that a very small but important group of women who go on to develop severe complications may potentially be over-represented among those with missing data.

Our findings provide further evidence to support the CMACE findings that additional efforts need to be made to support pregnant migrant women, particularly those who speak little or no English [10]. However, we additionally showed that certain ethnic groups are prone to late antenatal booking after English language ability and UK birthplace/non-UK birthplace were taken into account. Antenatal care may play an important role in establishing access patterns and gaining familiarity with the healthcare system in general; for those from migrant communities pregnancy may be the first time they access the healthcare in the UK.

The identification of groups at increased risk of late antenatal booking has important public health implications. Future research should focus on effective interventions to encourage these minority groups to engage with maternity services. The results of this study (as well as the wider study [11]) will be used to inform a package of interventions in East London aimed at improving early and consistent access to antenatal care.

## Conclusions

Our study demonstrates that maternal ethnicity, mother’s place of birth and ability to speak English are independently associated with antenatal booking later than 12 completed weeks of pregnancy in an ethnically diverse cohort in East London. Other key predictors for late antenatal booking include high parity and indicators of potential social vulnerability, such as living in temporary accommodation. Locally appropriate interventions should be designed to encourage these groups to engage with the maternity services early in pregnancy.

## Ethical approval

Ethical approval was obtained from the NHS Research Ethics Committee and UEL Ethics Committee prior to the study (Reference: 10/H0707/88; date: 20/09/2010).

## Abbreviations

NUHT: Newham University Hospital NHS Trust; NHS: National health service; GP: General practitioner.

## Competing interests

The authors declare that they have no competing interests.

## Authors’ contributions

JC analysed the data and wrote the first draft of the paper. GY took responsibility for data management and supported the analysis. BH supported the data extraction and cleaning process, developed the initial analytic plan and contributed to the interpretation of the analysis. JM was involved in drafting the initial protocol and liaised with staff at NUHT throughout the project. FJ contributed to the interpretation of the analysis. AH was the principal investigator and supervised the process. AR contributed to the development of the methodology; supervised the data extraction and cleaning process and supported development of the analytic plan. All authors provided comments on the paper. All authors read and approved the final manuscript.

## Pre-publication history

The pre-publication history for this paper can be accessed here:

http://www.biomedcentral.com/1471-2393/13/103/prepub
